# Supplementary material for: A Critical View of the Application of the APEX Software (Aqueous Photochemistry of Environmentally-Occurring Xenobiotics) to Predict Photoreaction Kinetics in Surface Freshwaters
Source: Molecules. 2019 Dec 18;25(1):9. doi: 10.3390/molecules25010009 (PMC7017383; doi:10.3390/molecules25010009)
Supplement: Supplementary file 1 [file molecules-25-00009-s001.zip › 3.2.4_gcc-4.4.0.zip!/3.2.4_gcc-4.4.0/3.2.4_gcc-4.4.0/doc/octave/html/interpreter/Ranges.html]

Ranges - Untitled


Next: Single Precision Data Types,
Previous: Matrices,
Up: Numeric Data Types


---

### 4.2 Ranges

A range is a convenient way to write a row vector with evenly
spaced elements. A range expression is defined by the value of the first
element in the range, an optional value for the increment between
elements, and a maximum value which the elements of the range will not
exceed. The base, increment, and limit are separated by colons (the
: character) and may contain any arithmetic expressions and
function calls. If the increment is omitted, it is assumed to be 1.
For example, the range

```
     1 : 5
```

defines the set of values [ 1, 2, 3, 4, 5 ], and the range

```
     1 : 3 : 5
```

defines the set of values [ 1, 4 ].

Although a range constant specifies a row vector, Octave does *not*
convert range constants to vectors unless it is necessary to do so.
This allows you to write a constant like 1 : 10000 without using
80,000 bytes of storage on a typical 32-bit workstation.

Note that the upper (or lower, if the increment is negative) bound on
the range is not always included in the set of values, and that ranges
defined by floating point values can produce surprising results because
Octave uses floating point arithmetic to compute the values in the
range. If it is important to include the endpoints of a range and the
number of elements is known, you should use the `linspace` function
instead (see Special Utility Matrices).

When adding a scalar to a range, subtracting a scalar from it (or subtracting a
range from a scalar) and multiplying by scalar, Octave will attempt to avoid
unpacking the range and keep the result as a range, too, if it can determine
that it is safe to do so. For instance, doing

```
     a = 2*(1:1e7) - 1;
```

will produce the same result as 1:2:2e7-1, but without ever forming a
vector with ten million elements.

Using zero as an increment in the colon notation, as 1:0:1 is not
allowed, because a division by zero would occur in determining the number of
range elements. However, ranges with zero increment (i.e., all elements equal)
are useful, especially in indexing, and Octave allows them to be constructed
using the built-in function ones. Note that because a range must be a row
vector, ones (1, 10) produces a range, while ones (10, 1) does not.

When Octave parses a range expression, it examines the elements of the
expression to determine whether they are all constants. If they are, it
replaces the range expression with a single range constant.
